# Long Noncoding RNAs in Metabolic Syndrome Related Disorders

**DOI:** 10.1155/2016/5365209

**Published:** 2016-11-02

**Authors:** Magdalena Losko, Jerzy Kotlinowski, Jolanta Jura

**Affiliations:** Faculty of Biochemistry, Biophysics and Biotechnology, Department of General Biochemistry, Jagiellonian University, Krakow, Poland

## Abstract

Ribonucleic acids (RNAs) are very complex and their all functions have yet to be fully clarified. Noncoding genes (noncoding RNA, sequences, and pseudogenes) comprise 67% of all genes and they are represented by housekeeping noncoding RNAs (transfer RNA (tRNA), ribosomal RNA (rRNA), small nuclear RNA (snRNA), and small nucleolar RNA (snoRNA)) that are engaged in basic cellular processes and by regulatory noncoding RNA (short and long noncoding RNA (ncRNA)) that are important for gene expression/transcript stability. In this review, we summarize data concerning the significance of long noncoding RNAs (lncRNAs) in metabolic syndrome related disorders, focusing on adipose tissue and pancreatic islands.

## 1. Introduction

**Figure 1:**
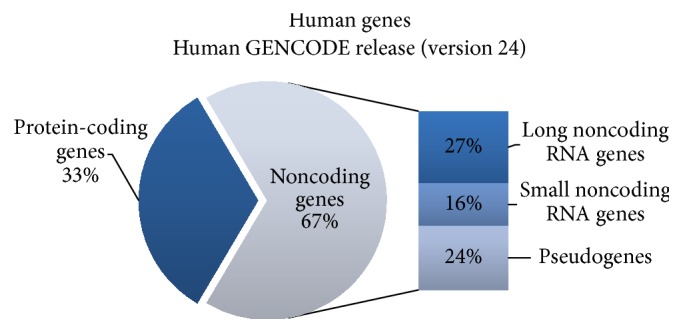
Schematic illustration of human genome presented by the Encyclopedia of DNA Elements Consortium (ENCODE). Detailed description of gene representation in the main text.

**Figure 2:**
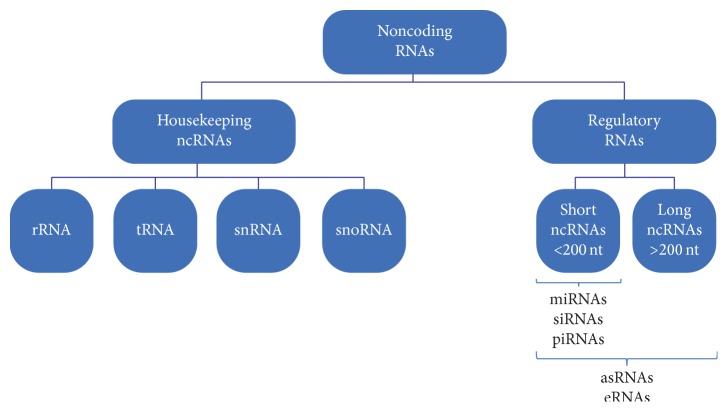
Types of noncoding RNAs (ncRNAs). Noncoding RNAs are classified into housekeeping and regulatory noncoding RNAs. Housekeeping ncRNAs include ribosomal (rRNA), transfer (tRNA), small nuclear (snRNA), and small nucleolar RNAs (snoRNAs). Regulatory noncoding RNAs are divided into short ncRNAs (<200 nucleotides) or long ncRNAs (>200 nucleotides) including microRNAs (miRNAs), small interfering RNAs (siRNAs), Piwi-associated RNAs (piRNAs), antisense RNAs (asRNAs), and enhancer RNAs (eRNAs).

Recent genome-wide transcriptome studies have revealed that the majority of the mammalian genome is transcribed, thereby giving rise to a range of coding and noncoding RNA (ncRNA) transcripts [1]. The latest Human GENCODE Release (version 24), presented by the Encyclopedia of DNA Elements Consortium (ENCODE), showed that the human genome is composed of 60554 genes (Figure 1). Among those, 19815 are protein-coding genes, whereas the remaining two-thirds of the genome represents noncoding RNA genes (15941 long noncoding RNA genes, 9882 small noncoding RNA genes, and 14505 pseudogenes). Noncoding RNAs are predominant in the genome and are classified as either housekeeping or regulatory noncoding RNAs. Housekeeping noncoding RNAs are constitutively expressed and they function as key regulatory molecules of many cellular processes. This group includes ribosomal (rRNA), transfer (tRNA), small nuclear (snRNA), and small nucleolar RNAs (snoRNAs). The second group, regulatory noncoding RNAs, is divided into short ncRNAs (<200 nucleotides) or long noncoding RNAs (lncRNAs, >200 nucleotides). Short ncRNAs are represented by microRNAs (miRNAs), small interfering RNAs (siRNAs), and Piwi-associated RNAs (piRNAs), and, in few cases, by antisense RNAs and enhancer RNAs (eRNAs). Long noncoding RNAs group includes antisense RNAs and enhancer RNAs (eRNAs) [1–3] (Figure 2).

**Figure 3:**
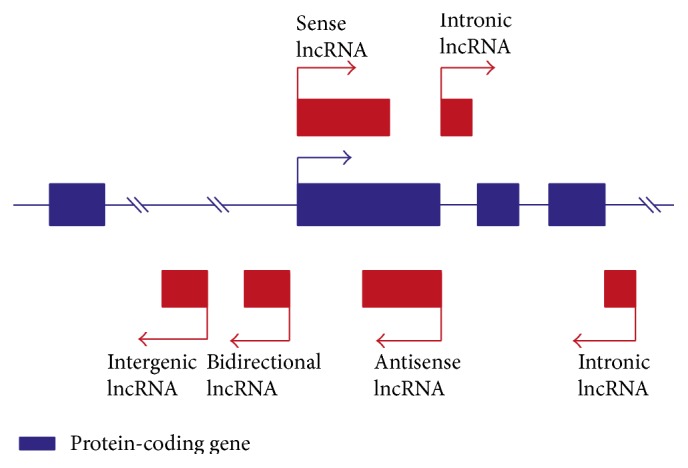
Classification of long noncoding RNAs. lncRNAs (shown as red boxes) may be classified into five categories: sense lncRNAs, antisense lncRNAs, bidirectional lncRNAs (transcribed on the opposite strand within 1 kb from the nearest protein-coding gene), intronic lncRNAs, and intergenic lncRNA (transcribed between protein-coding genes, but they are located at least 1 kb away from the nearest protein-coding gene) [3]. Detailed descriptions of lncRNA classes are in the main text.

In this review, we focus on the broadest class of ncRNA as represented by lncRNAs. This class of ncRNAs was first discovered in the mouse during large-scale sequencing of full-length cDNA libraries [4]. Generally, lncRNAs were characterized as transcripts longer than 200 nucleotides with some features typical for protein-coding mRNA. They are transcribed by RNA-polymerase (Pol) II [5], capped on the 5′ end, polyadenylated, and commonly expressed as alternatively spliced variants. Moreover, lncRNAs were found to have the same trimethylation marks of H3K4 and H3K36 at their promoters and transcribed regions [1]. However, they possess unique characteristics that distinguish them from mRNA. In contrast to mRNA, lncRNAs usually contain intron/exon structure, they are expressed at lower levels, often in a tissue-specific manner, and their sequence is poorly evolutionary conserved [6]. Additionally, they do not possess open reading frames (ORFs), 3′ UTR, and termination regions; thus the majority have limited coding potential. Nevertheless, recent studies have demonstrated that some lncRNAs can be associated with polysomes and ribosomes, suggesting that they may act as coding transcripts and give rise to small peptides [7]. An emerging number of studies related to the discovery of new lncRNAs and their position to the nearest protein-coding genes have resulted in the introduction of a new nomenclature for these molecules. Long noncoding RNAs may be classified into five categories: (1) sense lncRNAs that overlap the nearest protein-coding genes at the same strand; (2) antisense lncRNAs located across the exons of protein-coding genes from the opposite strand; (3) bidirectional lncRNAs transcribed on the opposite strand within 1 kb from the nearest protein-coding gene; (4) intronic lncRNAs that overlap intronic regions of coding genes in either the sense or antisense orientation; (5) intergenic lncRNAs that represent the largest group of lncRNA; they are located between protein-coding genes but are at least 1 kb away from the nearest protein-coding gene [3] (Figure 3).

## 2. Biological Function of Long Noncoding RNAs

In recent years, numerous studies have aimed to identify functions of newly discovered long noncoding RNAs. Although the primary role of lncRNAs was their epigenetic regulation of protein-coding gene expression, only a limited number of such long noncoding transcripts have been identified [21]. It was soon discovered that lncRNAs play a role in variety of biological process, acting in the nucleus, or in the cytoplasm, or in both [22]. Functions of lncRNAs are manifested by three types of interaction: RNA-RNA, RNA-DNA, and RNA-protein, all of which depend on the site of action [23].

In the nucleus, for example, the main role of lncRNAs is assumed to be epigenetic imprinting. One of the best-described mechanisms of lncRNAs action during the epigenetic regulation is the X chromosome inactivation, in which X chromosome inactive-specific transcript (XIST) plays a key role [24]. Recent studies demonstrated that lncRNAs can interact with chromatin remodeling complexes including polycomb repressive complex (PRC2) 2, Trithorax/MLL, and H3K9 methyltransferase G9a, acting as docking platforms to specific genomic loci [4]. These protein complexes modify DNA methylation patterns via trimethylation leading to transcriptional repression (PRC2) or activation (Trithorax/MLL) of the lncRNA target genes. Numerous lncRNAs have been recognized as scaffolds for inhibitory PRC2 including XIST, KCNQ1OT1, and HOTAIR [8, 25]. Other lncRNAs, like HOTTIP, directly bind to protein complexes of Trithorax/MLL [9]. In these situations, lncRNAs have been shown to drive or repress gene expression, respectively. Moreover, it has been postulated that these lncRNAs are guides for chromatin-modifying complexes, where they first recognize the target localizations at the chromatin, bind to them, and then form docking platforms for protein partners [26].

**Figure 4:**
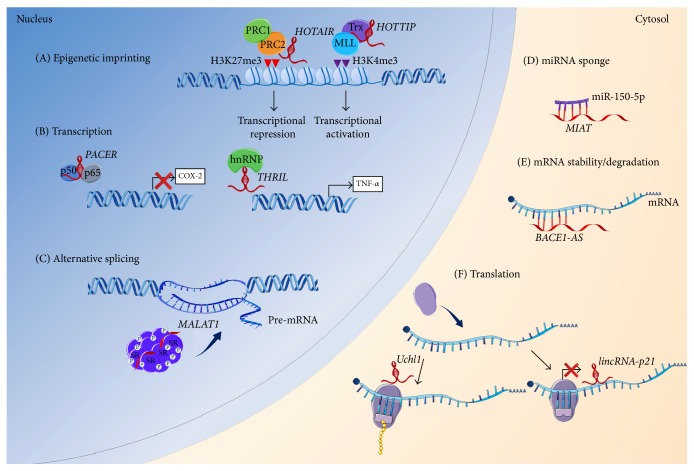
Biological role of lncRNAs. Role of lncRNAs has been implicated in the regulation of diverse processes, manifested by three ways of interactions: RNA-RNA, RNA-DNA, and RNA-protein, both in the nucleus and in the cytoplasm. (A) In the nucleus, lncRNAs have been shown to play a key role in imprinting control. lncRNAs may act as docking platforms for the chromatin remodeling complex, polycomb repressive complex (PRC2) 2 (e.g., HOTAIR), which methylates histone H3 at lysine 27 (H3K27me3) or with the heterogeneous group of Trithorax/MLL proteins (e.g., HOTTIP), leading to a repression or gain of transcriptional activity, respectively [8, 9]. (B) Other regulatory roles of lncRNAs in the nucleus include transcriptional regulation by interacting with transcription factors (e.g., PACER directly interacts with the repressive subunit of NF-*κ*B, p50, thus preventing it from binding to the Cox-2 promoter [10]), heterogeneous nuclear ribonucleoproteins (hnRNPs) (e.g., THRIL, together with hnRNPL, acts as RNA-protein complex and binds to TNF-*α* promoter and mediates the induction of TNF-*α* expression [11]). (C) Moreover, lncRNAs may regulate the pre-mRNA alternative splicing (e.g., MALAT1), which acts as scaffold for SR proteins, present in nuclear speckles and modulates their phosphorylation [12]. In the cytosol, lncRNAs exert their function by interacting with target transcripts and miRNAs through base-pairing. (D) For miRNAs, lncRNAs may act as a molecular sponge, preventing specific miRNAs from binding to their target mRNAs (e.g., MIAT binds to miR-150-5p, thereby upregulating the level of miR-150-5p target gene). (E) lncRNA binding to mRNA may stabilize (e.g., BACE1-AS prevents miRNA-induced repression of BACE1 transcript) or decay target transcripts. (F) Moreover, lncRNAs promote (e.g., antisense Uchl1 interacts with Uchl1 mRNA, resulting in recruitment of ribosomes [13]) or repress (e.g., lincRNA-p21, which binds to target mRNA, causing recruitment of translation repressors [14]) translation of transcripts. This figure was produced using modified elements from the Servier Medical Art (http://www.servier.com/).

In addition to regulation of genomic imprinting, lncRNAs are also involved in the control of gene expression at the transcriptional level. lncRNAs can directly interact with transcription factors, acting as decoys or inhibitors for their binding to the target DNA sequence. For example, two lncRNA molecules, Lethe and p50-associated Cox-2 extragenic RNA (PACER), have been shown to interact with different subunits of NF-*κ*B, thus preventing it from binding to the promoter region of the target gene [10, 27]. Moreover, lncRNAs exert their regulatory function during mRNA processing and stability, interacting with the heterogeneous nuclear ribonucleoproteins (hnRNPs). An example of this type of RNA-protein linkage is demonstrated for several lncRNAs including lincRNA-p21 [28], lincRNA-Cox2 [29], or THRIL [11]; thus they affect either activation or repression of target genes. Other actions of lncRNA in the nucleus are manifested by alternative splicing regulation [12] and subnuclear compartment formation [30] (Figure 4).

In the cytosol, lncRNAs exert their function by interacting with target mRNAs (or miRNAs) through base-pairing. In this manner, lncRNAs may either stabilize (e.g., BACE1-AS prevents miRNA-induced repression of BACE1 transcript [31]) or decay (e.g., 1/2-sbsRNAs bind to the Alu element of SMD target mRNAs within the 3′ UTR region [32]) target transcripts. Moreover, lncRNAs promote translation of transcripts (e.g., antisense Uchl1 interacts with Uchl1 mRNA, resulting in recruitment of ribosomes [13]) or repress this process (e.g., lincRNA-p21 binds to target mRNA causing recruitment of translation repressors [14]) (Figure 4).

## 3. Inflammation and Its Significance in Metabolic Syndrome

Inflammation is a complex process, aiming to defend the body against harmful agents by removing or neutralizing them in order to restore tissue homeostasis [33]. The complexity of this process results from the broad spectrum of inflammatory pathways, various inflammatory inducers, sensors, and mediators [34]. In the classic view, the inflammatory response is induced immediately after stimulation by the inflammatory stimuli, such as a bacterial pathogen [33]. The foreign antigen is recognized by the sensors of the host defense cell. Cells, such as neutrophils, dendritic cells (DCs), and macrophages, express evolutionarily conserved Toll-like receptors (TLRs) on their surface. The TLR family is composed of several classes of transmembrane proteins and recognizes specific structures present on the microbes, known as pathogen-associated molecular patterns (PAMPs) [35]. Induction of TLRs triggers a multistep signaling cascade, thereby resulting in the modulation of gene expression involved in the immune response. Key processes regulated by TLRs are related to the secretion of various mediators by immune cells, phagocytosis, cell migration, metabolic reprogramming, and tissue repair [36].

In general, based on the length, the inflammatory response can be classified into acute or chronic. Acute inflammation usually develops locally, and its role is to eliminate toxic agents, repair damaged tissue, and restore homeostasis. In some circumstances, local inflammation might be greater, therefore called a “generalized inflammatory response,” which is associated with the response of the whole organism [37]. Although the acute phase leads to the beneficial recovery of the injured tissue and healing, this nonspecific immune process could have also negative effects. Without the proper resolution phase, it can easily turn into a chronic state. Chronic inflammation may appear after the switch from the acute phase, and it is characterized by the specific, long-term cellular, and humoral immune response present at the site of tissue injury [38]. In consequence, it may significantly contribute to dangerous pathophysiological changes and initiate the development of chronic diseases.

Each inflammatory stage is subjected to precise control by several transcription factors involved in the immune response [39]. One such factor is NF-*κ*B (nuclear factor kappa-light-chain-enhancer of activated B cells), representing a family of transcription factors. NF-*κ*B plays an important role in gene expression regulation of multiple factors involved in immune, acute phase, and inflammatory responses [40]. NF-*κ*B, in addition to controlling the development and function of the immune system, is required in other physiological processes such as suppression of apoptosis and cell proliferation and differentiation. In mammals, the active form of NF-*κ*B is composed of homo- and heterodimers of the NF-*κ*B family members, which can be divided into five different subunits: p50, p52, p65 (RelA), c-Rel, and RelB. All of these share homology of the specific N-terminal domain responsible for the dimerization and binding to target DNA sequences. Depending on the local cytokines produced by the immune cells, NF-*κ*B also controls macrophage relocalization, activation, and differentiation into two different phenotypes: proinflammatory (M1) or anti-inflammatory (M2). In response to proinflammatory cytokines, NF-*κ*B is also involved in the activation of T lymphocytes, which proliferate and secrete cytokines such as interferon gamma (IFN*γ*), interleukin 6 (IL-6), or TNF-*α*. The result of NF-*κ*B activation is stimulation of proinflammatory cytokine production by macrophages and T lymphocytes, thereby enhancing the inflammatory response [41].

One disease closely linked to a chronic inflammatory state is obesity. It is characterized by an excessive fat accumulation and enhanced production of proinflammatory cytokines/chemokines by adipose tissues. It is commonly caused by a combination of excessive food intake, lack of physical activity, and genetic susceptibility [42]. A great majority of obese individuals present features of the metabolic syndrome: (a) increased waist circumference, (b) insulin resistance, (c) hyperglycemia, (d) hypertension, and (e) hypertriglyceridemia [15]. Appearance of these symptoms is associated with a high risk of serious disease development, including type 2 diabetes, cardiovascular diseases, [18], nonalcoholic fatty liver disease (NAFLD) [17], and even some cancers [19]. The mechanism of metabolic disorder development is still not fully understood; nevertheless numerous studies have suggested an adipocyte dysfunction as a main cause of metabolic system failure [43]. In this pathological state, adipose tissue secretes various mediators, including cytokines, inducing a chronic, low-grade inflammatory response that results in recruitment of immune cells into the tissue.

The excessive accumulation of tissue fat leads to disturbance both in the secretory and in the storage functions of adipocytes. It is postulated that the excessive fat mass induces the production of reactive oxygen species and the development of chronic oxidative stress in patients with obesity and metabolic dysfunction [44, 45]. In adipose tissue of obese patients is observed severe inflammatory response, characterized by high production of cytokines and increased infiltration of immune cells, including monocytes, stimulated to differentiate into macrophages and lymphocytes T, activated to proliferate and secrete cytokines [46, 47]. Cytokines secreted by activated macrophages act on TLRs present on the surface of adipocytes, stimulating them to produce adipokines and cytokines [48]. Based on these observations, it has been found that the reactions of both the immune and metabolic systems are closely connected, and they play an important role in the development of metabolic disorders.

## 4. lncRNA in Obesity and Adipogenesis Control

Adipocytes play a central role in energy homeostasis by fine-tuning the equilibrium between nutrient deposition (white adipose tissue, WAT) and energy expenditure (brown adipose tissue, BAT). Additionally, adipose tissue acts as an endocrine organ by secreting factors (adipokines) that regulate whole body energy and glucose homeostasis [49]. Adipogenesis is a complex process governed by a network of transcription factors, cofactors, and signaling intermediates from numerous pathways [50]. The transcriptional cascade that regulates adipocyte differentiation is predominantly driven by peroxisome proliferator-activated receptor gamma (PPAR*γ*), shown to be both necessary and sufficient for adipogenesis [51]. Importantly, adipogenesis is highly regulated not only by PPAR*γ* but also by the coordinated effects of other transcription factors including CCAAT/enhancer-binding proteins (C/EBPs), Kruppel-like factors (KLFs) or Wingless proteins (Wnt), and transcriptional cofactors [52–54].

**Figure 5:**
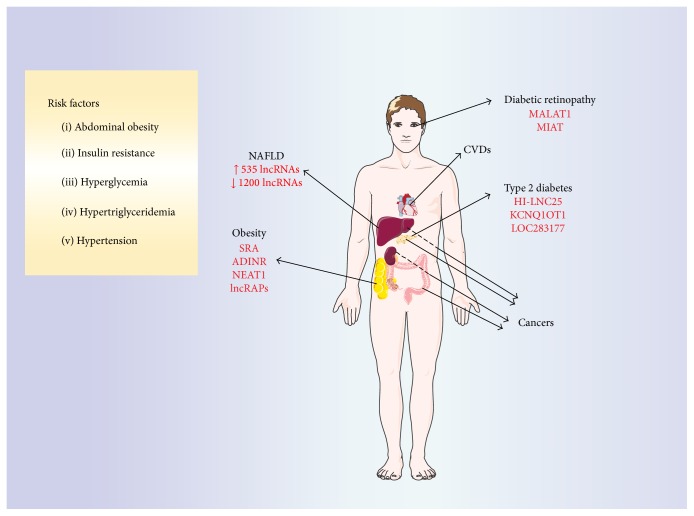
Development of metabolic syndrome. The common clinical symptoms of metabolic disorders include abdominal obesity, insulin resistance, hyperglycemia, hypertriglyceridemia, and hypertension [15]. Their appearance is associated with a high risk for development of serious diseases including type 2 diabetes, diabetes-related diabetic retinopathy [16], and nonalcoholic fatty liver disease (NAFLD) [17], as well as cardiovascular diseases (CVDs) [18] and even some cancers including liver, pancreas, and colon [19] (CVDs and cancers are not discussed in this review). Selected lncRNAs involved in the control of metabolic system activity (e.g., adipogenesis: SRA, ADINR, NEAT1, and lncRAPs) and pathology of metabolic diseases (e.g., type 2 diabetes, HI-LNC25, KCNQ1OT1, LOC283177, diabetic retinopathy, MALAT1, and MIAT) are marked with the red color. In NAFLD, 535 lncRNAs are upregulated and 1,200 are downregulated; however, these sequences are not yet well characterized [20]. This figure was produced using modified elements from the Servier Medical Art (http://www.servier.com/).

In recent years, there has been growing attention paid to noncoding RNAs as a novel, cell intrinsic regulatory mechanism. Specifically, lncRNAs are now emerging as important regulators of gene expression both on the transcriptional and on the posttranscriptional levels. Long noncoding RNAs are being discovered using modern techniques of microarray or next-generation sequencing. However, only small portions have been fully characterized. In the case of adipogenesis, there is wide expression of lncRNAs, controlling a variety of genes involved in the formation, differentiation, and activation of adipocytes (Figure 5).

Steroid receptor RNA Activator (SRA) lncRNA was described for the first time as a molecule regulating adipogenesis [55]. It was shown that lncRNA SRA, initially identified as a coactivator of steroid receptors, also acts as a transcriptional coactivator of PPAR*γ* [55, 56]. lncRNA SRA promotes adipocyte differentiation by binding to the N-terminal portion of PPAR*γ* and enhances its transcriptional activity. Additionally, its overexpression significantly increased mRNA and protein levels of adipocyte master regulators PPAR*γ* and C/EBP*α* as well as PPAR*γ* target genes. Conversely, knockdown of endogenous lncRNA SRA resulted in inhibition of 3T3-L1 preadipocyte differentiation. Moreover, gene expression profiling showed that lncRNA SRA regulates expression of genes related to various cellular processes including cell cycle and insulin transduction pathways [55]. Further experiments proved that lncRNA SRA overexpression inhibits phosphorylation of p38 mitogen activated protein kinase (MAPK) and c-Jun NH2-terminal kinase (JNK) in the early differentiation of ST2 mesenchymal precursor cells. In contrast, knockdown of lncRNA SRA increased p38 and JNK phosphorylation while reducing insulin receptor mRNA and protein levels, which led to decreased downstream signaling [57]. Supporting that data, SRA knock-out mice (SRA^−/−^) are resistant to high fat diet (HFD) induced obesity. After 14 weeks of HFD, SRA^−/−^ mice are characterized by reduced WAT mass and decreased expression of adipocyte genes such as adiponectin or fatty acid-binding protein 4. The lean phenotype is also associated with smaller adipocytes in WAT compared to WT mice, reduced liver mass, fewer lipid droplets in the liver, and decreased expression of lipogenesis-associated genes. Finally, SRA^−/−^ animals have improved insulin sensitivity [58].

In a recent paper, Xiao and coworkers [59] identified lncRNA ADINR (adipogenic differentiation induced noncoding RNA) that,* in cis*, transcriptionally activates C/EBP*α*. It was upregulated 20–30-fold during the course of adipogenesis and was transcribed from a position ~450 bp upstream of the C/EBP*α* gene. Knockdown of lncRNA ADINR with siRNA resulted in a dramatic adipogenic defect as shown by a decreased number of Oil Red O positive cells and reduced adipogenic transcripts C/EBP*α*, PPAR*γ*, fatty acid-binding protein 4, and lipoprotein lipase. Importantly, inhibition of adipogenesis caused by depletion of lncRNA ADINR was rescued by overexpression of C/EBP*α*. The lncRNA ADINR mechanism of action relies on its binding to PA1 followed by recruitment of MLL3/4 histone methyltransferase complexes. In turn, that causes an increase in H3K4me3 and a decrease in H3K27me3 histone modification in the C/EBP*α* locus during adipogenesis [59].

Another lncRNA that induces adipogenesis* in cis* was discovered within the mouse PU.1 locus. Expression of the PU.1 gene gives rise to both the mRNA encoding PU.1 protein and antisense (AS) lncRNA originating from a promoter in the antisense strand of intron 3 [60]. The protein PU.1 was originally demonstrated to be a key transcription factor regulating hematopoiesis, but recent studies revealed that it is also expressed in 3T3-L1 preadipocytes and murine adipocytes isolated from WAT [60, 61]. Gain and loss of function studies established the PU.1 protein as an inhibitor of 3T3-L1 preadipocyte differentiation, acting via downregulation of PPAR*γ* [62]. On the other hand, PU.1 AS lncRNA promotes adipogenesis through preventing PU.1 mRNA translation by binding to PU.1 mRNA to form an mRNA/AS lncRNA duplex [60]. Formation of this sense-antisense RNA duplex was also confirmed in porcine adipocytes by an endogenous ribonuclease protection assay combined with RT-PCR. In line with the previous data, expression of PPAR*γ* and fatty acid synthase (FASN) was significantly upregulated in the lncRNA PU.1 shRNA treated group [63].

In a number of studies, global transcriptome profiling was used to select specific lncRNAs either involved in adipogenesis or characteristic for adipocytes specifically isolated from WAT or BAT. In a seminal study done by Sun and coworkers [64], the transcriptome of primary brown and white adipocytes, preadipocytes, and cultured adipocytes was profiled. Scientists identified 175 lncRNAs that were specifically regulated during adipogenesis. To further validate lncRNAs that are functionally important for adipogenesis, researchers selected the top 20 lncRNA genes based on the following criteria: (i) significant upregulation in both brown and white fat cultures, (ii) binding to PPAR*γ* or CEPB*α* promoters, and (iii) independent validation of adipose-specific expression. By using RNAi-mediated loss of function, the top 10 most important lncRNAs were selected and called Regulated in AdiPogenesis (lncRAPs) in order to highlight their key role in proper differentiation of adipocyte precursors [64].

In comparison to white adipocytes, brown adipocytes have a higher mitochondrial content and express uncoupling protein 1 (UCP1), which disperses chemical energy through heat production. Brown adipose tissue helps to regulate energy expenditure in rodents and newborn babies, but it has been considered to have no physiologic relevance in adults [65, 66]. However, recent studies demonstrated that metabolically functional BAT is also present in adult humans. As a result, an understanding of BAT physiology might provide an effective treatment of obesity or other metabolic disorders [67]. Identification of the lncRNAs crucial for BAT differentiation and function was done using whole transcriptome RNA sequencing. Next, gain and loss of function studies established lncRNA Blnc1 as a potent activator of thermogenic adipocyte differentiation. It functions upon formation of RNA-protein complex with the early B cell factor 2 (EBF2) transcription factor to stimulate BAT formation. Additionally, lncRNA Blnc1 itself is a target of EBF2, thereby forming a feed-forward regulatory loop. On the other hand, despite its stimulatory effects on brown preadipocyte differentiation, lncRNA Blnc1 failed to promote differentiation of 3T3-L1 cells into UCP1 positive adipocytes [68]. Another global profiling of gene expression during mouse brown fat cell differentiation, followed by candidate lncRNAs genomic context analysis, pathway analysis, and gene ontology enrichment of their associated protein-coding genes was recently described. In this study, scientists identified three lncRNAs (Gm15051, Tmem189, and Cebpd) associated with their flanking coding genes (Hoxa1, C/EBP*β*, and C/EBP*δ*) that participated in adipose commitment [69].

More recently, RNA-seq analysis of murine brown, inguinal white, and epididymal white fat allowed for identification of lncRNA cluster enriched in BAT. Among them, the lncRNA BATE1 was induced during brown adipose tissue differentiation. Inhibition of BATE1 led to decreased expression of brown fat markers and mitochondrial genes. Authors showed that BATE1 binds to the heterogeneous nuclear ribonucleoprotein U, a factor also required for BAT formation [70].

Finally, interactions of lncRNAs with microRNAs in the context of adipogenesis were also described. As demonstrated by Gernapudi and coworkers [71], the miR-140/lncRNA NEAT1 signaling network is necessary for adipogenesis. Adipocyte derived stem cells isolated from miR-140 knock-out mice had dramatically decreased adipogenic capabilities found to be associated with the downregulation of lncRNA NEAT1 expression. Overexpression of NEAT1 in such cells was sufficient enough to restore their differentiation capabilities. Authors identified a miR-140 binding site in the NEAT1 sequence. They also found that the physical interaction with miR-140 in nucleus led to increased expression of NEAT1 [71].

Excessive fat accumulation not only causes pathological expansion of adipose tissue but also leads to triglyceride deposition in the liver. Presence of lipid droplets in excess of 5% of total hepatocytes is a diagnostic hallmark of nonalcoholic fatty liver disease (NAFLD) [72]. Although most patients suffer from a mild course of illness, approximately 25% of cases progress and can subsequently lead to the development of steatohepatitis, hepatic fibrosis, liver cirrhosis, and hepatoma [73]. Scientists and physicians estimate that between 20% and 30% of West's general population suffers from NAFLD, which makes this affects more than a billion people worldwide [74]. To address questions about the global expression pattern and functional contribution of lncRNAs during NAFLD, Sun and coworkers [20] analyzed microarray expression using RNA extracted from liver biopsies of healthy patients and those suffering from NAFLD. In this analysis, 535 lncRNAs were found upregulated in NAFLD samples compared with controls and 1,200 were downregulated in NAFLD (Figure 5). Out of these, seven NAFLD samples that had highly up- or downregulated gene expression were validated by quantitative real-time PCR (qPCR). Three lncRNA results obtained by qPCR were different in comparison to the microarray data, showing no change between analyzed groups. However, changes in other selected hits were confirmed, highlighting the importance of microarray data validation [20].

## 5. lncRNA in Diabetes and Pancreatic Function/Glucose Homeostasis

Pancreatic islets, through the secretion of endocrine hormones such as insulin and glucagon, play a key role in metabolic homeostasis. However, one of the most common malfunctions of *β*-cells is caused by lack of insulin, leading to the development of diabetes [75]. Additionally, diabetes may develop as a result of insulin resistance, a condition in which the body cannot effectively use the insulin produced by *β*-cells. As a result, blood sugar levels are deregulated and various tissues are exposed to prolonged hyperglycemia that, over time, causes serious damage to many of the body's systems. It should be emphasized that hyperglycemia, a hallmark of diabetes, can cause both acute and long-term complications like cardiovascular disease, stroke, foot ulcers, ketoacidosis, or hyperosmolar coma [76]. Based on etiology, WHO classifies diabetes into four categories, but the vast majority of cases fall into two broad etiopathogenetic groups: type 1 diabetes mellitus (T1DM) or type 2 diabetes mellitus (T2DM) [77].

Accounting for 5–10% of all cases, T1DM is characterized by the loss of insulin producing *β*-cells due to an autoimmune reaction. Autoimmune destruction of *β*-cells is unrelated to lifestyle. There are genetic variants known to increase the risk of T1DM development. Type 2 diabetes mellitus represents 90–95% of all cases and, unlike T1DM, is strongly associated with patients' lifestyle [78]. A number of lifestyle factors are known to be important for development of T2DM including obesity (BMI above 30), lack of physical activity, poor diet, or stress [79]. In fact, most patients suffering from T2DM are obese. It is a chronic disease that begins with insulin resistance, a condition in which cells (mostly adipocytes, hepatocytes, and muscle cells) fail to properly absorb and metabolize glucose. As a result, *β*-cells increase insulin production to overcome hyperglycemia, but this compensatory mechanism is transient and generally fails over time [77]. Intensified metabolism of *β*-cells leads to a gradual loss of their endocrine function and finally causes *β*-cell apoptosis. Similar to obesity, diabetes is a worldwide problem. According to WHO, in 2014, the global prevalence of diabetes was estimated to be 9% among adults aged over 18 years and it is expected to double before 2030 [75, 80]. Additionally, experts project that, in 2030, diabetes will be the 7th leading cause of death [75].

Initial studies aimed at identification of specific lncRNAs expressed in murine and human pancreatic islets were done in 2012 [81, 82]. By using next-generation sequencing, scientists revealed a large collection of 1359 intergenic lncRNAs expressed in murine *β*-cells (Figure 5). Many of them were highly tissue-specific, but their function was not evaluated, and future experiments are still needed [81]. Similar numbers of 1128 intergenic and antisense lncRNAs were identified in human *β*-cells and many of them (e.g., HI-LNC12 and HI-LNC77) were highly tissue-specific [82]. Additionally, lncRNA-encoding genes were preferentially located near genes encoding important regulators of *β*-cell function, development, and transcription. One of them, lncRNA HI-LNC25, conserved between mouse and human, was shown to positively regulate GLIS3 mRNA. Silencing of lncRNA HI-LNC25 by shRNA depleted GLIS3 mRNA. GLIS3 encodes a pancreatic islet transcription factor that is mutated in monogenic diabetes and contains T2DM risk variants [83, 84]. In the same set of experiments, scientists also showed that selected lncRNAs (e.g., HI-LNC12 and HI-LNC25) are linked to *β*-cell differentiation program as their expression was significantly higher in human islets when compared to the embryonic pancreas. Moreover, the addition of glucose to* in vitro β*-cell culture induced expression of some lncRNAs, suggesting their importance for mature islet cell physiology. Finally, authors showed deregulation of islets' lncRNAs in T2DM by comparing profiles of lncRNAs in islets from 19 nondiabetic and 16 T2DM donors. Two lncRNAs, namely, KCNQ1OT1 and HI-LNC45, were significantly increased or decreased in T2DM islets, respectively [82].

Seven lncRNAs specific for *β*-cells, as reported by Morán and coworkers [82], were also discussed in another study that analyzed pancreatic islets from 89 individuals with or without diabetes. Microarray analysis, RNA sequencing, and exome sequencing methods enabled researchers to identify 493 lncRNAs that were differentially expressed in the pancreatic islets. Out of those, 17 long intergenic noncoding RNAs (lincRNAs) were significantly associated with donor's HbA1c levels, and two (LOC283177 and SNHG5) were also involved in gene expression regulation. To obtain insight into functional target genes of lncRNAs LOC283177 and SNHG5, the authors performed a coexpression analysis, linking their expression with all other genes in pancreatic islets. lncRNA LOC283177 levels correlated with expression of genes that play a key role in islet function, namely, synaptotagmin 11, MAP-kinase activating death domain (MADD), and paired box 6 (PAX6). Synaptotagmin 11 is known to regulate the exocytosis of insulin and MADD proinsulin synthesis, and* PAX6* is involved in development of pancreatic islets [85, 86]. Supporting these findings, lncRNA LOC283177 expression was found to be directly associated with insulin exocytosis in the islets [87].

Insulin is secreted from *β*-cells in response to glucose, while other nutrients such as free fatty acids and amino acids can augment glucose-induced insulin secretion. In addition, various hormones (e.g., leptin, growth hormone, glucagon like peptide-1, and estrogen) also regulate insulin secretion. As a result, *β*-cells are called a metabolic hub in the body that coordinate nutrient metabolism with endocrine system [88]. Insufficient insulin production, or insulin resistance, is a prime cause of diabetic complications. Among the many tissues affected by hyperglycemia, endothelial cells are very important because they are implicated in pathogenesis of diabetes-related microvascular and macrovascular complications [16].

One of these complications, diabetic retinopathy, was recently shown to be influenced by lncRNA MALAT1 (metastasis-associated lung adenocarcinoma transcript 1) [89] (Figure 5). In a mouse model of streptozotocin-induced diabetes, scientists performed lncRNA expression profiling and compared expression between diabetic and control retinas. Microarray analysis, followed by qPCR validation of selected genes, allowed for identification of 303 differentially expressed lncRNAs including 214 downregulated and 89 upregulated genes in diabetic versus nondiabetic samples. One gene, lncRNA chr19: 5795689–5802671, a murine ortholog of human lncRNA MALAT1, had a greater than 10-fold upregulation in diabetic retinas [89]. lncRNA MALAT1 was previously described in humans to be deregulated in several solid tumors and was associated with cancer metastasis and recurrence [90]. It was shown to be significantly upregulated in a RF/6A cell model of hyperglycemia, in the aqueous tumor samples and in fibrovascular membranes of diabetic patients [89].

To further investigate the molecular mechanism of lncRNA MALAT1 action, scientists decided to analyze its function in retinal vasculature and endothelial cell dysfunction in diabetes mellitus [91]. lncRNA MALAT1 knockdown in retinas of db/db mice resulted in amelioration of diabetic retinopathy as manifested by reduced apoptosis of retinal cells and pericytes. Additionally, retinal inflammation was also alleviated. Importantly, after administration of lncRNA MALAT shRNA, scientists observed decreased retinal vascular permeability as measured by Evans blue leakage. Moreover,* in vitro* tests showed that lncRNA MALAT1 knockdown decreased retinal endothelial cell proliferation, migration, and tube formation. In RF/6A endothelial cells, lncRNA MALAT1 acts via induction of p38 MAPK signaling, and its silencing reduced phosphorylated p38 level but had no effect on phosphorylated ERK1/2 or JNK1/2. Pretreatment of RF/6A cells with SB203580, a p38 MAPK pathway inhibitor, strongly blocked the effect of lncRNA MALAT1-induced cell proliferation [91].

More recently, the same research group reported that lncRNA MIAT (myocardial infarction-associated transcript) also regulates diabetes mellitus microvascular dysfunction, acting as a competing endogenous RNA [92] (Figure 5). Similar to a previous report, lncRNA MIAT expression was increased in diabetic retinas and endothelial cells cultured in high glucose medium. As shown by* in vivo* tests, lncRNA MIAT knockdown ameliorated diabetes mellitus induced retinal microvascular dysfunction. In line with* in vivo* data, silencing of lncRNA MIAT in endothelial cells cultured* in vitro* led to an inhibition of proliferation, migration, and tube formation. lncRNAs may act as an mRNA molecular sponge and regulate miRNAs available for binding their target mRNAs. Bioinformatics suggested that the lncRNA MIAT sequence contains 4 putative miRNA binding sites including miR-29a-3p, miR-29b-3p, miR-29c-3p, and miR-150-5p. One of these, miR-150-5p, was proven to directly target lncRNA MIAT in endothelial cell both* in vitro* and* in vivo* [92]. This particular miRNA is of great importance for angiogenesis because it regulates the expression of VEGF, a key angiogenic factor involved in physiological and pathological angiogenesis [93]. Yan and coworkers [92] proved that MIAT regulates the expression of miR-150-5p target gene VEGF creating a critical regulatory loop for endothelial cell function. During angiogenesis, lncRNA MIAT is significantly upregulated, which in turn alleviates the miR-150-5p repression effect, thereby upregulating the level of miR-150-5p target gene, VEGF [92].

In contrast to lncRNA MIAT, lncRNA MEG3 (maternally expressed gene 3) expression was significantly downregulated in the retinas and endothelial cells of STZ-induced diabetic mice upon high glucose and oxidative stress conditions [94]. Its knockdown regulated retinal endothelial cell proliferation, migration, and tube formation* in vitro*. As demonstrated in RF/6A endothelial cells, inhibition of lncRNA MEG3 by siRNA significantly increased levels of phosphorylated PI3K and phosphorylated Akt at Thr^308^ and Ser^473^. However, levels of the total PI3K and total Akt were not affected. Addition of PI3K inhibitors to cell cultures abrogated the observed phenotype, proving that lncRNA MEG3 regulates hyperproliferation of retinal endothelial cells through PI3K/Akt signaling [94]. PI3K/Akt signaling is an important signaling pathway regulating glycogen metabolism. Additionally, its activation influences endothelial cell biology by modulating angiogenesis, proliferation, and microvascular permeability [95].

## Additional Points

*Summary*. The data demonstrated above highlights an important role of newly identified lncRNAs in the etiology and progression of metabolic diseases. Importantly, new reports about lncRNAs, such as the identification of lncRNA GAS5 whose serum levels correlate with T2DM, aid in leading to a better understanding of this complex processes [96]. However, further cell culture and animal studies are needed to fully clarify lncRNA's mechanism of action. Our understanding of lncRNA function is still very limited and important questions need to be addressed. First, how is their expression regulated? Taking into account that many lncRNAs are expressed in a cell type specific manner, their expression must be tightly regulated. As a result, currently available databases of lncRNAs are likely to be incomplete. Secondly, little is known about the biological functions of lncRNAs. Despite large numbers of differentially expressed molecules in various diseases, only a few have been thoroughly studied. For* in vitro* studies, although there are many utilizable techniques (e.g., RNA-seq, crosslinking immunoprecipitation, or chromatin isolation by RNA purification), we still lack a proper* in vivo* model. Thirdly, very little is known about secondary and tertiary structures of lncRNAs. Such studies might help to better predict lncRNA function or enable a more detailed classification. Finally, as mentioned previously for lncRNA GAS5, lncRNAs may serve as a prognostic or diagnostic marker. However, the reliability and sensitivity of such markers require careful evaluation.
